# GPS tracking of free-roaming dogs and human spillover risk of *Echinococcus granulosus* in highly endemic Peru

**DOI:** 10.3389/fvets.2025.1647590

**Published:** 2025-11-18

**Authors:** Katherine Morucci, Lizzie Ortiz Cam, Elvis W. Diaz, Guillermo Porras-Cotrina, Javier Bustos, Manuela Verastegui, Cesar M. Gavidia, Ricardo Castillo-Neyra

**Affiliations:** 1 School of Veterinary Medicine, University of Pennsylvania, Philadelphia, PA, United States; 2 One Health Unit, School of Public Health and Administration, Universidad Peruana Cayetano Heredia, Lima, Peru; 3 Center for Global Health, Universidad Peruana Cayetano Heredia, Lima, Peru; 4 Infectious Diseases Laboratory Research–LID, Faculty of Sciences and Philosophy, Universidad Peruana Cayetano Heredia, Lima, Peru; 5 School of Veterinary Medicine, Universidad Nacional Mayor de San Marcos, Lima, Peru; 6 Department of Biostatistics, Epidemiology and Informatics, Perelman School of Medicine at University of Pennsylvania, Philadelphia, PA, United States

**Keywords:** animal movement, dog, *Echinococcus granulosus*, GPS tracking, one health, spatial analysis, tapeworm

## Abstract

**Introduction:**

Cystic echinococcosis (CE), a neglected disease that results from infection with the larval stage of the *Echinococcus granulosus sensu lato (s.l.)* tapeworm, poses significant zoonotic risk to humans and is a persistent threat in developing agricultural communities around the world. While the prevalence of human CE in the central highlands of Peru has previously been estimated around 5–7%, true prevalence is likely higher given the protracted period of asymptomatic disease, reduced medical access of at-risk populations, increased contact between herders, livestock, and herding dogs, and poor understanding of local disease epidemiology. To beIer understand CE epidemiology in a highly endemic region of Peru, we studied the movement of free-roaming dogs in the community of Chanchayllo, Junin, Peru.

**Methods:**

We performed copro-ELISA to identify *E. granulosus s.l.* positive dogs, tracked the ranging behavior and calculated home ranges of 19 owned, free-roaming dogs to understand the movement of the definitive host of *E. granulosus s.l.* on the landscape. Specifically, we investigated the spatial association between *E. granulosus*-infected dog home ranges and proximity to their owners’ houses and a local slaughterhouse.

**Results:**

*Echinococcus granulosus s.l.* infection prevalence was alarmingly high in our canine population, with 85% positivity (binomial exact 95% CI: 62.1–96.8%). All dog home ranges overlapped with their owners’ households, and notably, even negative dog households overlapped with nearby positive dog home ranges.

**Discussion:**

These data suggest that widespread environmental contamination of *E. granulosus s.l.* egg-containing feces may be a significant driver of locally elevated disease prevalence in human populations. We use our findings to understand the local disease ecology of CE in free roaming dogs, assess pillover risk, and guide future intervention strategies aimed at reducing human cases. Our findings suggest that existing trategies delivering anthelmintic drugs to individual households have the potential to reduce spillover of *E. granulosus s.l.*

## Introduction

Free-roaming dogs play a critical role in the ecology of *Echinococcus granulosus sensu lato (s.l.)*, the parasitic tapeworm that causes cystic echinococcosis (CE) in humans and animals. CE is a zoonotic disease that poses a significant threat to human and animal health, with canines serving as definitive hosts and livestock as intermediate hosts (1, 2). Infection remains a significant public health concern in regions that engage in livestock production, such as the central Andes in Peru (3). Previous studies have emphasized the prevalence of CE in regions like Junin due to the close proximity and interaction between dogs and livestock, which are central to the economy and agricultural practices of these areas (4, 5).

*Echinococcus granulosus* has an indirect life cycle which requires infection of multiple hosts to successfully survive from an egg to sexual maturity. The reproductive stage of *E. granulosus* parasitizes the gastrointestinal tract of its definitive host, wild and domestic canids [CDC-DPDx (6–8)]. Eggs shed in feces are highly environmentally stable and may persist outside of a host for extended periods of time (8, 9). Parasite eggs hatch upon consumption by an intermediate host, migrate into the bloodstream, and become lodged in viscera where parasite larvae contribute to slow-growing cystic lesions [CDC-DPDx (6, 8, 10)]. To complete its life cycle, *E. granulosus* larva must be consumed by scavenging canids feeding upon infected offal. While herbivorous species such as sheep tend to serve as the intermediate host, unfortunately, humans that consume *E. granulosus s.l.* eggs may also become infected and develop cysts in various organs, such as the lungs and liver (11–13). Without medical screening, CE may go undetected for years, or until cysts grow large enough to contribute to life-threatening disease that warrants surgical intervention (4, 14–17). That said, surgical interventions may not be readily available to the most vulnerable populations due to reduced medical access in developing agricultural economies (18–20). The persistence of CE in endemic regions poses a serious threat to human health and wellbeing. Halting transmission requires a clear understanding of animal reservoirs, environmental conditions that favor parasite persistence, and human behaviors that facilitate spillover. This challenge calls for a truly One Health approach—where animal, human, and environmental health overlap and where integrated interventions are most urgently needed (21).

The district of Canchayllo, in Junin, Peru, is a rural region located in the Central Andes. This region is known for its agricultural economy, and specifically, many families in this district engage in livestock management. The interdependence between sheep, herders, and herding dogs is central to this lifestyle. Unfortunately, these conditions provide a favorable environment for the transmission of *E. granulosus s. l.* Previous studies have estimated the prevalence of human CE in the central highlands of Peru at 5–7% based on ultrasound-based population surveys (3, 19). However, we believe that prevalence is likely higher given the protracted period of asymptomatic disease, reduced medical access of at-risk populations, high degree of contact between herders, dogs, and their livestock, and poor understanding of local disease epidemiology (19, 22).

Little is known about the contribution of free-roaming dogs to the epidemiology of CE in the Central Andes in Peru due to the limited number of integrative research initiatives bridging human and veterinary health. This study sought to better understand *E. granulosus s.l.* spillover risk, defined here as the transmission of parasite eggs from infected dogs to humans as an accidental, dead-end host, by examining parasite prevalence and ranging behavior in free-roaming dogs. While we refer to this phenomenon as spillover throughout the manuscript, we acknowledge that this term is usually used to describe cross-species transmission events involving biological barriers that prevent sustained transmission. In this case, humans serve as a dead-end host as they are susceptible to infection upon exposure, but do not permit subsequent infection of other hosts. Our goal was to establish an environmentally focused understanding of regional features that may be contributing to local disease transmission. To this end, we tracked free-roaming dogs to understand the movement of the definitive host of *E. granulosus* on the landscape and performed copro-ELISA to identify *E. granulosus s.l.* positive animals.

Most studies on echinococcosis in dogs have focused on working dogs. In contrast, our study aimed to better understand pet dogs, which may also play an important role in transmission to humans. Therefore, our first aim was to estimate the intensity of space use (e.g., probability of dog presence in specific areas) to inform spatially-targeted dog interventions. Specifically, we investigated the spatial association between *E. granulosus s.l.*-infected dog home ranges and proximity to their owners’ houses and a local slaughterhouse. In addition, knowing that free-roaming dogs often defecate along the borders of their territory to establish a “property line” (23), our second aim was to estimate the home range and core areas used by these dogs as a proxy for the spatial extent of spillover risk (e.g., transmission from dog feces to humans). We use our findings to increase our understanding of free-roaming dog ecology and spillover risk to inform interventions aimed at reducing human infection.

## Methods

### Study area

This study was conducted from June to July of 2023 in the district of Canchayllo, Jauja, Junin, Peru. Chanchayllo is one of the 34 districts in the province of Jauja and is located in the Central Andes within the Nor Yauyos Cochas Landscape Reserve. The district covers an area of 974.69 km^2^ and is located at 3,800 masl. The climate is cold, with temperatures ranging between 6 and 20 degrees Celsius.

This region is home to 1,601 inhabitants, most of whom are engaged in livestock farming, and specifically, sheep herding (24). The interdependence between humans, their dogs, and sheep herds associated with agrarian lifestyles not only provides a favorable environment for the completion of the *E. granulosus s.l.* life cycle, but also for the accidental spillover into human populations. Moreover, suspected and actual case data from nearby medical centers between 2019 and 2022 suggest that up to 31% of the total population within this district exhibits clinical signs compatible with CE symptomatology (unpublished data). This suggests prevalence in this area may be much higher than previously published findings in endemic regions (3, 19). We decided to conduct our study in two communities within this highly endemic district, given the unique occupational and lifestyle hazards presented to herders and their family members and suspected case prevalence.

**Figure 1:**
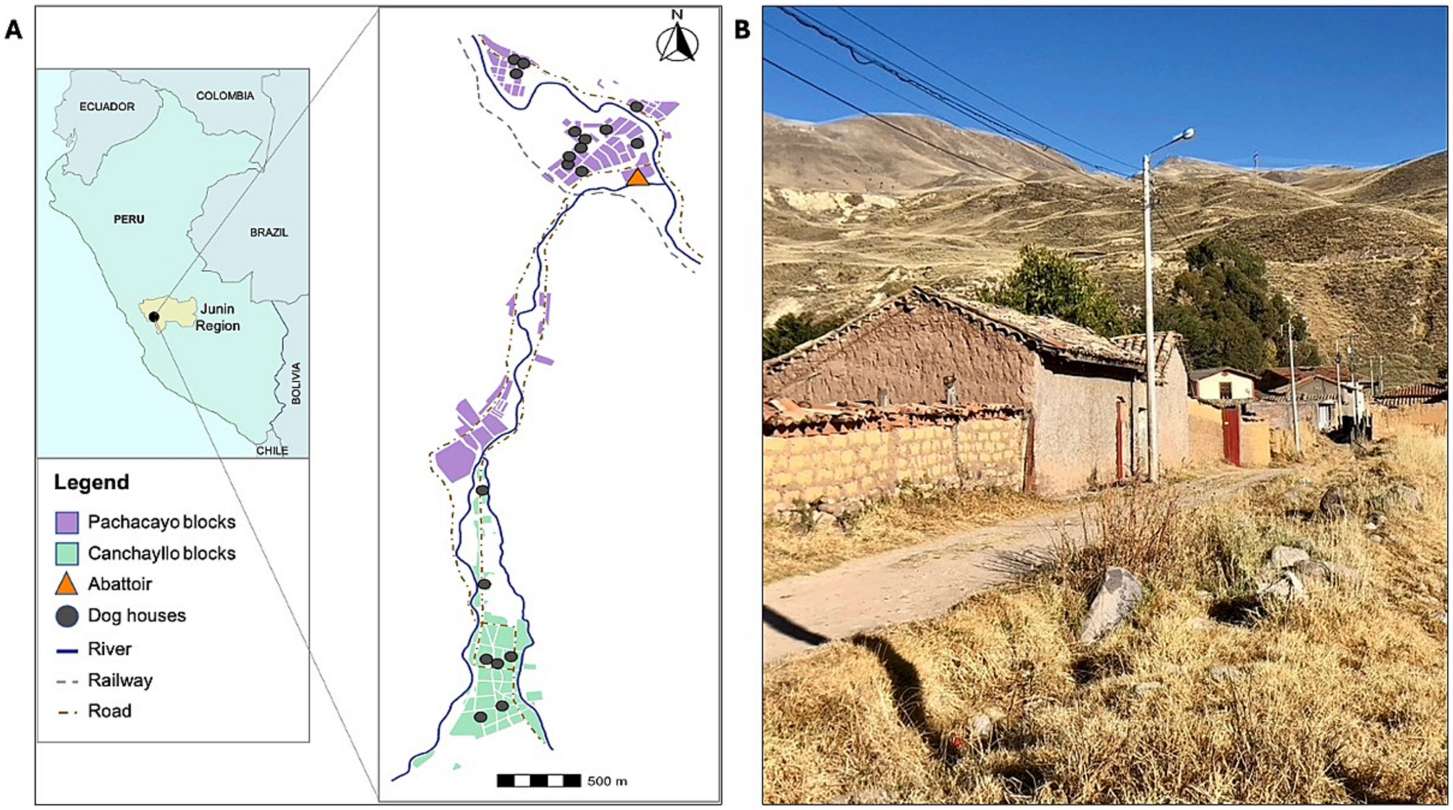
Map of the study region demonstrating **(A)** the location of the District of Canchayllo, Jauja - Junin, within the central Andes of Peru, and a street view showing the interconnectivity between the two study sites, the communities of San Juan de Pachacayo and Canchayllo, and their relation to the Pachacayo slaughterhouse and participant households, and **(B)** a photograph of a street containing houses in the community of Canchayllo.

**Figure 2:**
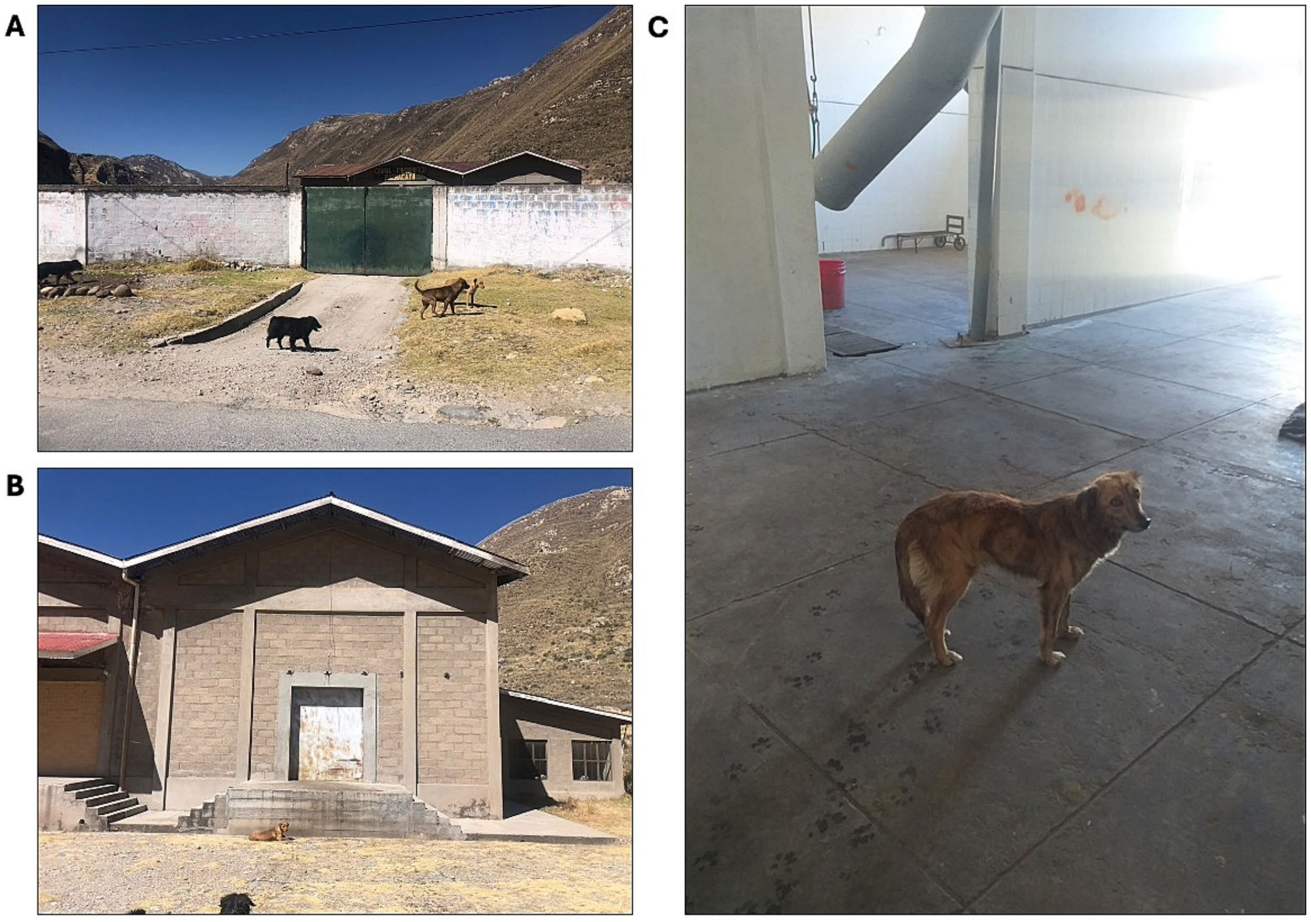
Images of the Pachacayo slaughterhouse which permits access to scavenging free-roaming dogs. Despite the presence of an external barrier **(A)**, dogs are often found on the inside of the wall **(B)** due to the gate being left open during operation hours, and can readily access the facility from entrances at the back of the property. Moreover, dogs are frequently found throughout the facility where all animal processing takes place **(C)**, where we have observed them consuming discarded offal.

Two sites within the district of Canchayllo were selected for inclusion in this study: the communities of San Juan de Pachacayo and Canchayllo (Figure 1). The selection of each site was based on proximity to a regional slaughterhouse that does not have appropriate barriers to prevent canine access (Figure 2), and thus may serve as a potential source of contaminated offal for free-roaming dogs. Moreover, the presence of a local river served as a geographical barrier that we predicted would permit dog movement between the study sites while limiting significant East–West displacements. We hypothesized that (1) free-roaming owned dogs associated with households near the slaughterhouse in the community of Pachacayo would be more likely to be infected with *E. granulosus s.l.* than dogs associated with homes in the community of Canchayllo, and (2) that the home ranges of *E. granulosus*-infected dogs will overlap with a majority of households in the study communities, representing a high risk of parasite spillover.

### Selection and recruitment

We identified potential participants based on the vicinity of their homes to the Pachacayo slaughterhouse and the presence of free-roaming dogs outside or near their household. Specifically, we attempted to balance participation from households located in the communities of Pachacayo and Canchayllo to determine if the presence of the slaughterhouse influenced dog movement and infection. We then conducted abbreviated door-to-door interviews to identify a subset of animals that were owned but spent the majority of their time free-roaming outside of the owner’s household. Owners were informed of study objectives, agreed to have feces collected from their pet, and to have the location of their dog tracked and mapped over the study period of 10 days. We recorded the sex, age, and physical characteristics of enrolled dogs and collected brief information about the degree of interaction between the dog and any other animals or livestock kept in or near the house. We did not intend to identify individual-level factors associated with positivity status or ranging behavior, and thus we prioritized inclusion based on location of owner’s homes and dog demographic features were unbalanced. Exclusion criteria included dogs that were 6-months of age or younger, small-bodied breeds/dogs, and animals with restricted access to the outdoors. After identifying a subset of dogs to enroll, we asked owners to restrain their animals while we placed a GPS-embedded collar around their necks.

### Data collection

We identified 20 dogs to enroll in our study. Dogs were manually restrained by their owners to be fitted with cloth collars containing a Mobile Action i-gotU GT-600 GPS logger. Based on our previous work (25), GPS loggers were programmed to record GPS coordinates of the animal at 3-min intervals. Dogs were visited daily to monitor the GPS tracker battery levels and the quality of recorded data. One dog was immediately lost to follow up due to the removal of the collar and tracking device, but collars remained in place and devices were successfully retrieved from the other 19 animals included in this study. While collar retrieval times varied between 7 to 10 days, GPS loggers were occasionally found turned off and thus recording times varied from 3 to 10 days.

### Fecal sample management and Copro-ELISA analysis

Approximately 2 grams of fresh feces were collected from each of our study animals. After asking owners about their dogs’ elimination routines, our team waited for each animal to defecate to collect their feces. If a dog was not observed defecating during the initial visit, we conducted multiple follow-up visits to avoid the use of canine purgative treatments. In the field, fecal samples were saved and preserved in PBS formalin and potassium dichromate and maintained at room temperature during transport to Laboratorio de Epidemiología y Economía Veterinaria, Facultad de Medicina Veterinaria, Universidad Nacional Mayor de San Marcos, Lima, Peru. The time between collection and arrival at the lab varied between 2 to 10 days. To identify positive samples in the lab, we implemented a validated sandwich copro-ELISA technique based on the capture and detection of somatic *E. granulosus* antigens (26). A cut-off point was selected as the highest value for both sensitivity and specificity by using ROC curves over the already known infected and non-infected dog stool samples.

### Spatial data analysis

The GPS data collected was downloaded as csv files from the backend of the iGotU application and exported into the statistical software R^1^ for further analysis and cleaning.

For each dog, we used two distinct computational methods to calculate dog home ranges (HRs) and core areas based on their abilities to address our aim of identifying areas that may present elevated spillover risk to owners based on dog movement and infection status. First, we generated HRs and core areas based on the minimum convex polygon (MCP) approach using the adehabitatHR package in R. To determine the most appropriate stable HR level for our analyses, we plotted 80–100% MCP HR levels for all dogs(S1). We found that most dogs’ HRs were stable through the 95% level, and that ranging behavior beyond this point was highly variable. As such, HRs were calculated by mapping the smallest polygon that fit 95% of each dog’s nearest ranging data points. The furthest 5% of ranging points for all dogs were excluded as random visitations. The MCP polygons were used to estimate HR areas and to calculate spatial variables concerning HR geometry, such as HR centroids. HR centroids were then used to measure distances between each animal’s HR and structures of interest, such as their owner’s household and the slaughterhouse. Core areas, which reveal where dogs spend the majority of their time, were calculated using the 50% isopleth.

We also calculated 95 and 50% isopleths using the kernel utilization distribution (KUD) approach to determine areas where dogs spend the greatest proportion of their time within their HR. This technique assesses the density of ranging data points and can distinguish between regions where a dog is most likely to be found at any given point in time, from areas that are more transiently occupied, such as those used to traverse a core area or HR. We used this approach, and specially the 50% isopleth, to identify where a dog would most likely be encountered for targeted interventions.

### Statistical analysis

Non-parametric analyses were performed to identify associations between infection status, dog demographic data, HR size and distances between HRs and areas of interest. All further statistical analyses were conducted in R.

### Ethics

IACUC ethical approval was obtained from Universidad Peruana Cayetano Heredia (approval number: 67258) and the University of Pennsylvania (approval number: 806514).

## Results

### Copro-ELISA

We collected 20 total fecal samples. Of those samples, 18 were collected from dogs included in our study, but we were unable to observe one of the collared dogs defecate during our time in the field. Two additional samples were taken from dogs that were found frequently ranging alongside dogs in our study. Overall, 17 of 20 dogs (85%; binomial exact 95% CI:62.1–96.8%) were copro-ELISA positive (Table 1).

**Table 1:** Ranging behavior and home range sizes of dogs in Canchayllo, Junin, Peru.

Dog ID	Sex	Age (y)	Community	Infected	95% MCP HR size (km^2^)	95% KUD HR size (km^2^)	50% MCP Core Area size (km^2^)	50% KUD Core Area size (km^2^)	Distance 95% MCP HR centroid and home (m)	Distance 95% MCP HR centroid and slaughter-house (m)
1	M	7	Pachacayo	1	1.000	1.092	0.514	0.045	691.77	338.17
2	F	6	Canchayllo	1	0.020	0.029	0.002	0.002	13.37	2546.80
3	F	4	Pachacayo	0	0.037	0.036	0.003	0.003	30.00	461.48
4	M	1	Pachacayo	1	1.644	1.830	0.307	0.196	639.25	441.09
5	M	2	Canchayllo	1	0.329	0.166	0.010	0.009	74.44	4105.04
6	M	2	Canchayllo	0	0.707	1.385	0.050	0.217	81.10	4521.32
7	M	1	Canchayllo	1	4.810	3.806	0.660	0.446	1735.44	5311.12
8	M	1	Canchayllo	1	0.258	0.208	0.009	0.012	155.58	3200.84
9	M	1	Pachacayo	1	0.401	0.422	0.035	0.040	136.76	228.61
10	M	2	Pachacayo	1	0.175	0.099	0.009	0.007	151.04	186.50
11	M	1	Pachacayo	1	0.048	0.097	0.004	0.004	36.80	346.62
12	M	3	Pachacayo	1	1.975	1.637	0.342	0.138	880.29	629.44
13	F	4	Pachacayo	1	0.097	0.128	0.011	0.002	80.97	369.89
14	M	2	Canchayllo	0	0.048	0.043	0.003	0.003	12.05	3738.54
15	M	2	Pachacayo	1	0.259	0.168	0.009	0.007	68.40	246.60
16	M	2	Pachacayo	1	0.123	0.076	0.006	0.007	58.22	149.49
17	M	5	Pachacayo	1	0.024	0.032	0.002	0.001	1.58	1001.82
18	M	7	Canchayllo	1	0.014	0.016	0.001	0.001	18.46	3673.71
19	M	1	Pachacayo	1	0.026	0.042	0.003	0.005	95.30	1001.73

MCP, Minimum convex polygon; KUD, Kernel utilization distribution; HR, Home range.

### Dog ranging analysis

**Figure 3:**
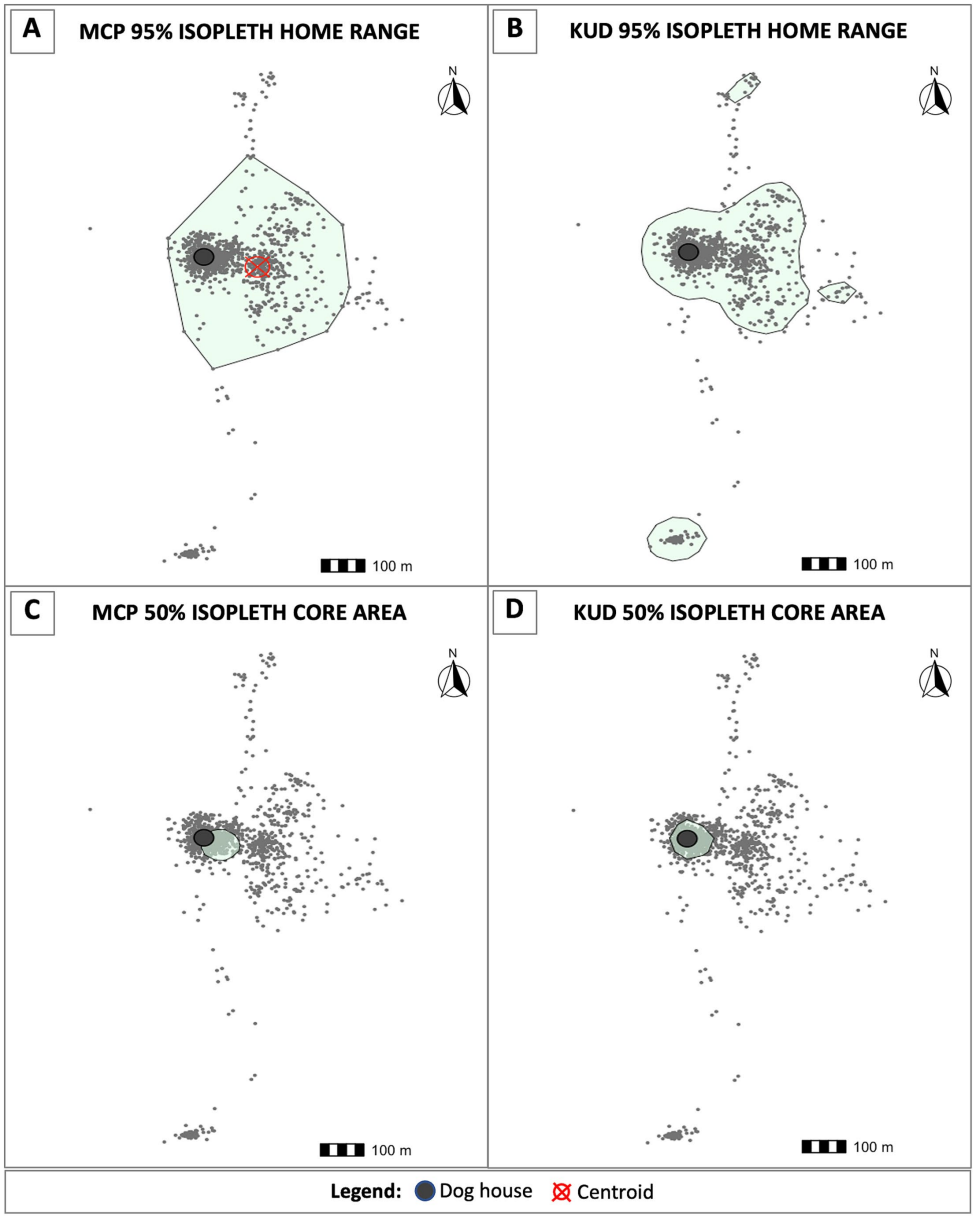
Home ranges and core areas for each dog were estimated using two distinct methods. **(A)** The minimum convex polygon (MCP) approach was implemented to calculate the smallest polygon that encapsulates the nearest 95% of ranging data points. Using the 95% MCP home ranges, we calculated centroids (red cross-hatch) to measure distances from the weighted center of each dog’s home range to their owner’s home (dark gray circles) and the slaughterhouse. We also estimated home ranges using the kernel utilization distribution (KUD) approach. The 95% KUD home ranges were used to determine where a dog is most likely to be found throughout their home range, and as a proxy to determine risk of environmental contamination due to the probability of encountering egg-containing feces on the landscape **(B)**. Core areas were calculated using the 50% MCP **(C)** and KUD **(D)** isopleths, respectively.

Using the MCP approach, we estimated HRs areas for each dog in our study (Figure 3). We calculated the weighted centroid for each dog’s HR, which was then used to determine the distances between animals’ HRs, their owners’ household, and the Pachacayo slaughterhouse (Table 1). The average distance between a dog’s HR and household is 289.74 m, which is significantly less than the average distance between HRs and the slaughterhouse, 1720.46 m (Mann–Whitney U test: *p* = 2.192e-05). There was only a single case in which a dog’s MCP core area did not overlap or border their owner’s home, indicating that 18/19 dogs in our study spend the vast majority of their time near home. In addition, only seven dogs had HRs that overlapped with the slaughterhouse. The association between HRs that overlap with the slaughterhouse and *E. granulosus s.l.* infection status was insignificant (Fisher’s test: *p* = 0.26). Finally, HR size did not influence infection status (Mann–Whitney U test: *p* = 0.07332). No individual-level dog demographic factors were statistically associated with positivity status or ranging behavior.

**Figure 4:**
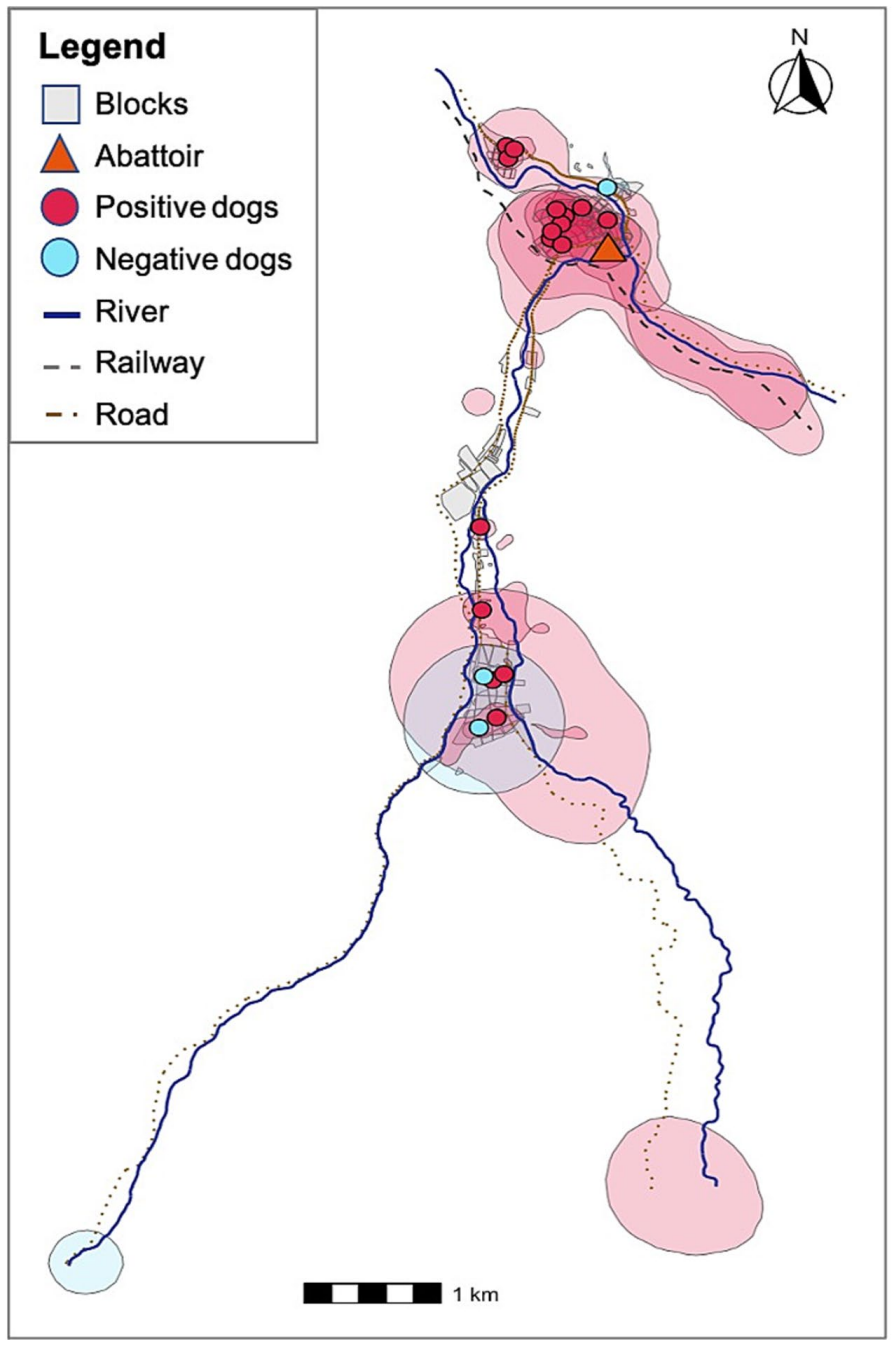
KUD dog home ranges and their households colored by *E. granulosus-s.l.* infection status. Red home ranges indicate *E. granulosus s.l.*-positive animals and blue home ranges correspond to negative animals. All households are overlapped by positive dog home ranges.

Using the MCP approach, all dogs’ home ranges overlapped with their owners’ households as well as with many other houses in the communities. To explore the potential threat of free-roaming dogs on *E. granulosus* spillover to humans, we estimated the geospatial spread of KUD HRs across the study region. Home ranges generated using the KUD function provide a more accurate model of spatial utilization behavior in that they distinguish between areas where dogs commonly spend time from the paths that animals use to traverse between areas of interest. In other words, the KUD-generated HRs provide a better estimation of risk areas. We overlaid copro-ELISA positivity status on KUD HRs to create a visual risk map depicting *E. granulosus s.l.*-positive dogs HRs, or areas that are likely contaminated with feces from infected animals (Figure 4). Notably, the HRs of the 16 infected animals overlap with all participant households in the study region. This suggests that there is a significant risk of spillover to individuals living in these communities based on environmental contamination.

## Discussion

*Echinococcus granulosus s.l.* infection prevalence was alarmingly high in our canine population, with 85% positivity. Importantly, the total area covered by *E. granulosus*-infected dog home ranges overlap with all households in the study communities. This is a remarkable finding considering that we only sampled 20 dogs and suggests that widespread environmental contamination of *E. granulosus* egg-containing feces may be a significant driver of local disease persistence and a high exposure risk for individuals living in this area. Although dogs tend to defecate repeatedly in specific locations within their territory (23), contamination is not necessarily limited to these areas. Several studies have shown that *Echinococcus* eggs are highly resistant in the environment and can be dispersed passively by biotic and abiotic vectors, such as wind and coprophagous insects (27–29). These mechanisms allow egg dissemination in a radial pattern from the original site of deposition, potentially expanding the contamination zone beyond the dog’s most heavily used areas. This highlights both the importance of considering both direct deposition sites and secondary dispersal routes when assessing environmental risk.

Our observations of the slaughterhouse revealed that, despite a perimeter wall and gate, it remained accessible to free-roaming dogs through open entrances and rear access points. A few dogs were seen consuming discarded viscera inside the premises, with no formal system for offal disposal or personnel restricting access. Thus, the facility represents a potential source of infection. But we found no significant association between infection status, home range size, or proximity to the slaughterhouse. In fact, dogs in our study rarely visited the slaughterhouse, and when they did, these visits did not coincide with operational hours, suggesting that this facility is unlikely to be a major source of infection. However, many families in the study region do not rely on the slaughterhouse, and instead slaughter livestock near their homes, a practice often accompanied by feeding discarded offal deemed unfit for human consumption to dogs (30–32). This pattern is consistent with our findings, as all dog home ranges overlapped with owners’ households, and even those with the smallest home ranges that do not overlap with the slaughterhouse were positive. Although our questionnaire did not directly ask about at-home slaughter, informal conversations with participants and other community members, as well as direct field observations, confirmed that this is a widespread practice in both study communities. This risky behavior likely plays an important role in the high observed prevalence, yet remains largely unaddressed by health programs in Peru.

Previous intervention strategies to control *E. granulosus* transmission in the central highlands of Peru, including Junín, have primarily stemmed from government-led mass treatment campaigns focused on periodic deworming of dogs using praziquantel (33–36). These efforts were often accompanied by educational messaging discouraging the feeding of offal to dogs and encouraging improved slaughterhouse hygiene (37, 38). However, such campaigns have faced significant limitations, including inconsistent implementation due to limited resources, lack of follow-up, low community participation, and limited integration of veterinary and public health services (39). In many rural communities like Canchayllo, access barriers and limited engagement of local stakeholders have further weakened the effectiveness of these interventions (40, 41). Given our findings—particularly the persistence of infection in dogs and their frequent movement between households and communal areas—these past strategies have been insufficient to disrupt the parasite’s life cycle. A more sustainable and targeted approach is urgently needed, including enhanced surveillance and context-specific education campaigns grounded in a One Health framework (42).

An important finding of this study was that the HRs of all dogs and all but one dog’s core area overlap with their households, indicating that they spend most of their time near their owners’ homes. This information supports that existing intervention strategies based on delivering anthelmintic drugs to individual households have the potential to reduce the environmental presence of infectious *E. granulosus* eggs. Delivering deworming agents to dog owners would offer a simple solution to ensure that dogs receive the necessary preventative care to curtail the zoonotic cycle that places owners at increased risk of infection. Given that dogs are often found near home, there would be plenty of opportunities for public health inspectors and dog owners to find the dogs and to offer them consumable preventative treatment at necessary intervals to provide protection against reinfection. While this would not address pre-existing environmental contamination, it would be a critical step in reducing further spillover risk to humans.

Our study has several strengths, but also some limitations. While we distributed our sampling across the different study communities, we did not have access to a household roster to conduct true random sampling. Additionally, the tracking period for animals varied and was limited to less than a week. It is possible that seasonal events (e.g., livestock fairs) influence movement patterns in ways that could affect *E. granulosus s.l.* transmission. We did not use purgatives, which may have increased the chances of false negatives; however, given the high prevalence we observed, we believe this did not significantly affect our results. Finally, we did not explore in detail the practice of home slaughtering by dog owners, which may help explain some of our findings.

Our findings suggest that in areas where *E. granulosus s.l.* is highly endemic (43), humans may face a high risk of infection regardless of the infection status of their own dog due to environmental contamination and overlap by nearby positive, free-roaming dog home ranges. Additionally, dogs experience high infection rates regardless of direct access to slaughterhouses, highlighting the importance of informal and home-based slaughtering practices. The consistent overlap between dogs’ core areas and their owners’ households underscores the potential for household-targeted interventions. Our findings support other studies that propose integrated control strategies that go beyond slaughterhouse management and include regular deworming of dogs, safe disposal of livestock offal, community education on zoonotic transmission routes, and improved surveillance of canine infections. Targeting interventions at the household and community level is likely to be essential for reducing human risk and breaking the transmission cycle of this neglected but persistent zoonosis. Targeted interventions could be informed by the spatial ecology of free-roaming dogs, which is closely intertwined with human behavior, the environment, and livestock management systems. While our study contributes valuable data to the understanding of free-roaming dog ecology, it represents only one component of a broader, complex system. Additional research is essential to build a comprehensive evidence base capable of supporting integrated, One Health–oriented approaches to the control of this persistent zoonotic disease.

## Data Availability

The original contributions presented in the study are publicly available. This data can be found here: https://doi.org/10.5281/zenodo.17081023.

## Footnotes

^1^https://www.R-project.org, version 3.4.0
