# Supplementary figures and images for: GPS tracking of free-roaming dogs and human spillover risk of Echinococcus granulosus in highly endemic Peru
Source: Front Vet Sci. 2025 Nov 18;12:1647590. doi: 10.3389/fvets.2025.1647590 (PMC12673269; doi:10.3389/fvets.2025.1647590)

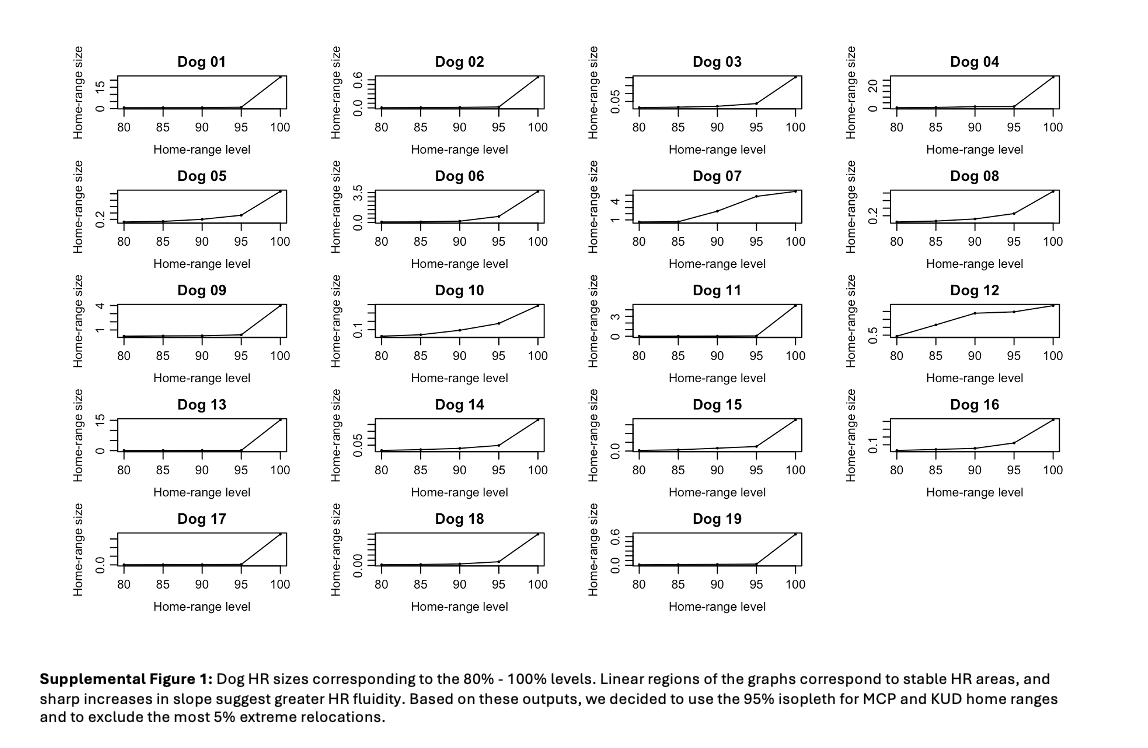

Supplement: Supplementary file 1 [file Image_1.png]
